# A critical evaluation of TRPA1-mediated locomotor behavior in zebrafish as a screening tool for novel anti-nociceptive drug discovery

**DOI:** 10.1038/s41598-019-38852-9

**Published:** 2019-02-20

**Authors:** Mee Jung Ko, Logan C. Ganzen, Emre Coskun, Arbaaz A. Mukadam, Yuk Fai Leung, Richard M. van Rijn

**Affiliations:** 1 Department of Medicinal Chemistry and Molecular Pharmacology, College of Pharmacy, West Lafayette, USA; 2 Department of Biological Sciences, College of Science, West Lafayette, USA; 3 Purdue Institute for Integrative Neuroscience, West Lafayette, USA; 4 Purdue Interdisciplinary Life Sciences Graduate Program, West Lafayette, USA; 5 Purdue Institute for Drug Discovery, West Lafayette, USA; 6 Department of Biochemistry and Molecular Biology, Indiana University School of Medicine, West Lafayette, IN 47907 USA

## Abstract

Current medications inadequately treat the symptoms of chronic pain experienced by over 50 million people in the United States, and may come with substantial adverse effects signifying the need to find novel treatments. One novel therapeutic target is the Transient Receptor Potential A1 channel (TRPA1), an ion channel that mediates nociception through calcium influx of sensory neurons. Drug discovery still relies heavily on animal models, including zebrafish, a species in which TRPA1 activation produces hyperlocomotion. Here, we investigated if this hyperlocomotion follows zebrafish TRPA1 pharmacology and evaluated the strengths and limitations of using TRPA1-mediated hyperlocomotion as potential preclinical screening tool for drug discovery. To support face validity of the model, we pharmacologically characterized mouse and zebrafish TRPA1 in transfected HEK293 cells using calcium assays as well as *in vivo*. TRPA1 agonists and antagonists respectively activated or blocked TRPA1 activity in HEK293 cells, mice, and zebrafish in a dose-dependent manner. However, our results revealed complexities including partial agonist activity of TRPA1 antagonists, bidirectional locomotor activity, receptor desensitization, and off-target effects. We propose that TRPA1-mediated hyperlocomotion in zebrafish larvae has the potential to be used as *in vivo* screening tool for novel anti-nociceptive drugs but requires careful evaluation of the TRPA1 pharmacology.

## Introduction

Nociception plays an active role in the defense against injury; however, persisting pain may become maladaptive and significantly impact an individual’s daily activity and the quality of life. Chronic pain, defined as unrelieved and persistent, lasting longer than 3 months, is usually treated by non-steroidal anti-inflammatory drugs (NSAIDs), anticonvulsants, tricyclic antidepressants, and opioids. Despite these treatment options, many patients still complain that their pain is insufficiently managed^1^. Additionally, opioid-based therapeutics have recently been demoted to third and fourth line treatment options for chronic pain per the prescription guidelines of the Center for Disease Control and Prevention due to their addictive potential, thereby further limiting the number of effective therapies. Thus, a critical need exists to identify novel pain targets and develop better analgesics for chronic pain.

An untapped analgesic target for chronic pain is the Transient Receptor Potential subfamily A1 (TRPA1) channel^2,3^. TRPA1 channels are calcium-permissive cation channels targeted by thermal^4,5^, mechanical^6,7^, and noxious chemical stimuli such as allyl isothiocyanate (AITC), acrolein, cinnamaldehyde, allicin, and formalin^8–10^. Pharmacological inhibition of TRPA1 channels inhibited complete Freund’s Adjuvant (CFA)-induced mechanical allodynia in wild-type mice, but not in TRPA1-deficient mice^6^. Oral administration of the TRPA1 antagonist, HC-030031, increased paw withdrawal threshold in a spinal nerve ligation model of neuropathic pain^11^. Yet, drug development targeting TRPA1 is still in its infancy, and thus far no TRPA1 ligand has been approved by the Food and Drug Administration. This may be in part because using the rodent models to establish *in vivo* efficacy of drug candidates can be very expensive and time-consuming.

The limitations associated with using a mouse model early in the drug discovery process motivated us to search for an alternative animal model that could expedite the process of validating *in vivo* TRPA1 ligand efficacy. Zebrafish have long been used as a preclinical vertebrate model organism for testing pharmacodynamics (absorption, distribution, metabolism and excretion), and pharmacokinetics of novel drugs^12^. The low cost, rapid development and high fecundity of zebrafish makes it ideal as a drug-screening tool. Several behavior models of neurological and neuropsychiatric-like behavior have been created in zebrafish that mimic those established for rodents, such as conditioned place preference^13^ and anxiety-like behavior^14^. Increased zebrafish locomotor behavior has also been previously observed by both thermal and chemical activation of TRPA1 channels^15,16^. Fortunately, TRPA1 channels are relatively conserved across species ranging from planarians to humans^17^, and the peripheral and central nociceptive systems of zebrafish are similar to many vertebrates such as mice and humans^18–20^. However, in slight contrast to humans and rodents, the zebrafish genome encodes two TRPA1 genes: *trpa1a*, and *trpa1b* (which will be called zTRPA1a and zTRPA1b in this study)^21^. To establish TRPA1 agonist-induced zebrafish hyperlocomotor activity as drug screening tool, it is important to characterize the pharmacology of TRPA1 agonists and antagonists between these two paralogs.

We hypothesize that hyperlocomotion induced by the activation of zebrafish TRPA1 can serve as a phenotypic screen for novel anti-nociceptive drug discovery. To address our hypothesis, we investigated if locomotor behavior of zebrafish larvae adheres to TRPA1 channel pharmacology. We measured calcium influx of TRPA1 channels in HEK293 cells transiently expressing mouse TRPA1, zebrafish TRPA1a, or zebrafish TRPA1b in response to TRPA1 ligands. The mouse TRPA1 pharmacology in HEK293 cells and nocifensive behavior in mice were also examined upon TPRA1 activation to support the face validity of the zebrafish model. Finally, we evaluated dose-dependent changes of nocifensive swimming behavior in zebrafish larvae following the exposure to TRPA1 ligands.

## Results

### Two TRPA1 agonists have similar potency but different kinetics to mouse TRPA1

**Figure 1:**
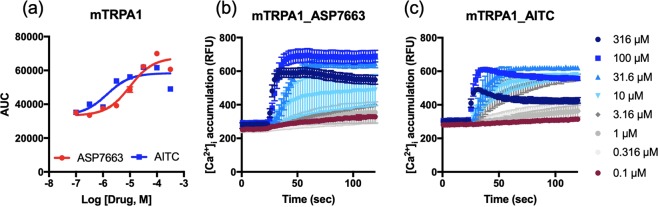
Potency of TRPA1 agonists (ASP7663 and AITC) in mTRPA1-transfected HEK293 cells. (**a**) Dose-response curve of ASP7663 and AITC in mTRPA1-transfected HEK293 cells. (**b**,**c**) Relative Fluorescent Unit (RFU) of ASP7663 and AITC was measured to plot the dose response calcium influx in (**a**).

To test previously known TRPA1 channel agonists, we produced and analyzed dose-response curves of ASP7663 and AITC in mouse TRPA1 (mTRPA1)-transfected HEK293 cells. The potency of the two agonists were measured based on area under the curve (AUC) of individual calcium accumulation in Fig. 1b,c. The dose-response curve of ASP7663 and AITC indicated that AITC and ASP7663 displayed similar potency in mTRPA1 (Fig. 1a, ASP7663: pEC_50_ = 5.16 ± 0.16, 6.8 µM, n = 8; AITC: pEC_50_ = 5.24 ± 0.3, 5.8 µM, n = 5; unpaired t-test *p* = 0.8106). The recorded potency for ASP7663 is ~10-fold weaker than previously reported in a similar FLIPR-based calcium assay^22^. Interestingly, we noticed that at 316 µM ASP7663 produced a more persistent calcium influx compared to AITC up to 120 seconds (Dark blue line, Fig. 1b,c). The calcium influx around 40–50 seconds was particularly decreased upon application of AITC in mTRPA1 (Fig. 1c). Furthermore, we have also found that ASP7663 could elicit calcium responses in non-transfected HEK293 cells with ASP7663. The potency for this unknown off-target effect was pEC_50_ = 4.27 ± 0.03 (54.1 µM n = 3), and thus a log unit lower than the TRPA1 response (Supplemental Fig. 1). The off-target calcium response also appeared to have slower kinetics similar to AITC (Compare Fig. 1c with Supplemental Fig. 1b).

### TRPA1 antagonists inhibit ASP7663-induced calcium influx to mTRPA1

**Figure 2:**
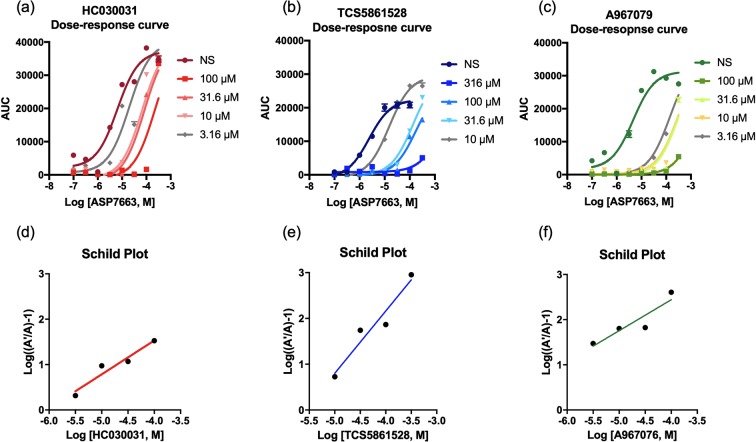
TRPA1 antagonists dose-dependently attenuate ASP7663-mediated TRPA1 calcium influx in mTRPA1-transfected HEK293 cells. (**a**–**c**) Representative dose-response curve of TRPA1 agonist ASP7663 in the absence or presence of three TRPA1 antagonists, HC-030031, TCS-5861528, and A-907076. Note the shift in the dose response curve of ASP7663 towards the right with increasing concentration of the antagonist. (**d**–**f**) Schild plot for the three TRPA1 antagonists HC-030031, TCS-5861528, and A-967076. Representative curves are shown.

The majority of TRPA1 studies use AITC to activate TRPA1 channels. However, given the unique kinetics observed in Fig. 1c and its potential ability to activate other TRP channels^23,24^, we decided to use the TRPA1 agonists ASP7663, which is supposedly more selective^22^. First, we chose to assess the ability of the TRPA1 agonist ASP7663 and the TRPA1 antagonists HC-030031, TCS-5861528, and A-967076 to induce or prevent calcium mobilization, respectively. HEK293 cells transiently expressing mTRPA1 channels were exposed to the TRPA1 agonist ASP7663 and intracellular calcium levels were fluorescently measured using the FLIPR-based calcium assay. The TRPA1 antagonists, HC-030031 and TCS-5861528, which are structurally very similar, shifted the ASP7663 dose-response curve towards the right in a dose-dependent manner (Fig. 2a,b). The pA_2_ of HC-030031 was 5.65 ± 0.2 (2.2 µM, n = 4, Fig. 2d), and the pA_2_ of TCS-5861528 was 5.34 ± 0.2 (4.6 µM, n = 4, Fig. 2e), indicating similar antagonist-channel affinities. A-967076 also shifted the ASP7663 dose-response curve towards the right (Fig. 2c), and the pA2 of A-967076 was 7.0 ± 0.3 (0.09 µM, n = 3, Fig. 2f). We found that HC-030031 and TCS-5861528 exhibit lower antagonist-channel affinities than A-967076 (One-way ANOVA, F_2,10_ = 12.52, *p* = 0.0019, HC-030031 vs. A-967076 *p* = 0.0094, TCS-5861528 vs. A-967076 *p* = 0.0024 with Tukey’s multiple comparison). Individual calcium traces for ASP7663 in the presence of the antagonists are presented in Supplemental Fig. 2. We did not observe any calcium influx with HC-030031 in non-transfected HEK293 cells (Supplemental Fig. 3a,b) or an off-target effect in mTPRA1-transfected HEK293 cells (Supplemental Fig. 3c,d). These results validate the use of ASP7663 to activate mTRPA1 to induce a calcium response and confirm the affinity of the TRPA1 antagonists for mTPRA1.

### HC-030031 blocked ASP7663-induced mechanical hypersensitivity in mice

**Figure 3:**
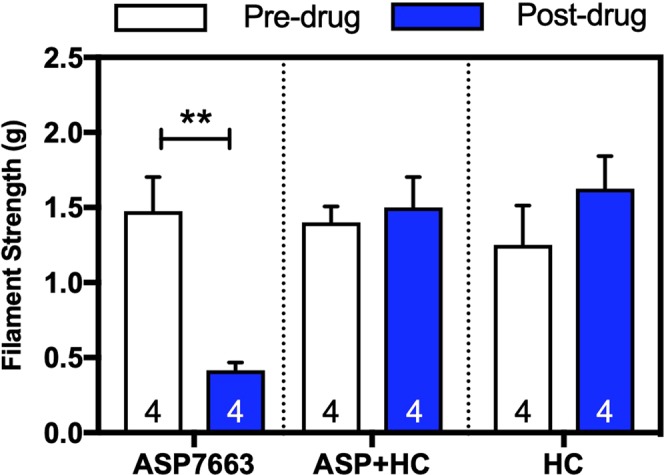
HC-030031 blocked ASP7663-induced mechanical hypersensitivity in C57BL/6 mice. Mechanical sensitivity was measured in C57BL/6 mice pre- and post-drug administration (n = 4 per treatment) in response to von Frey filament stimulation. Systemic administration of HC-030031 (100 mg/kg, p.o.) blocked mechanical hypersensitivity induced by ASP7663 (1 mg/kg, i.p.). (Two-way ANOVA, Effect of pre-drug x post-drug: F_1,18_ = 1.522, *p* = 0.2332; Group effects: F_2,18_ = 4.426, *p* = 0.0273; After Bonferroni’s multiple comparison, Pre-ASP7663 vs. Post-ASP7663: *p = 0.0034).

Having confirmed that the TRPA1 compounds are functional *in vitro*, we next determined whether TRPA1 activation using ASP7663 would result in a painful response and whether this effect would be blocked by a TRPA1 antagonist in C57BL/6 mice. Intraperitoneal administration of a previously determined effective dose of ASP7663 (1 mg/kg) significantly decreased mechanical thresholds of von Frey filaments as evaluated by two-way ANOVA, (Fig. 3, Effect of pre-drug x post-drug: F_1,18_ = 1.522, *p* = 0.2332; Group effects: F_2,18_ = 4.426, *p* = 0.0273). Bonferroni’s multiple comparison further indicated statistical significance between pre- and post-drug mechanical thresholds in the mice administered with ASP7663 (*p* = 0.0034), suggesting that ASP7663 administration increased mechanical sensation and nocifensive behavior. Based on a previous study^22^, we used an oral administered dose of HC-030031 (100 mg/kg) to block the ASP7663-mediated mechanical hypersensitivity (*p* > 0.99). Bonferroni’s multiple comparison did not show a statistical significance between pre- and post-drug mechanical thresholds in the mice administered vehicle (*p* = 0.8692 after unpaired t-test, Supplemental Fig. 4) or HC-030031 (*p* = 0.97 after Bonferroni multiple comparision, Fig. 3).

### ASP7663 and AITC have similar potency to two zebrafish TRPA1 paralogs but lower potency than mTRPA1

**Figure 4:**
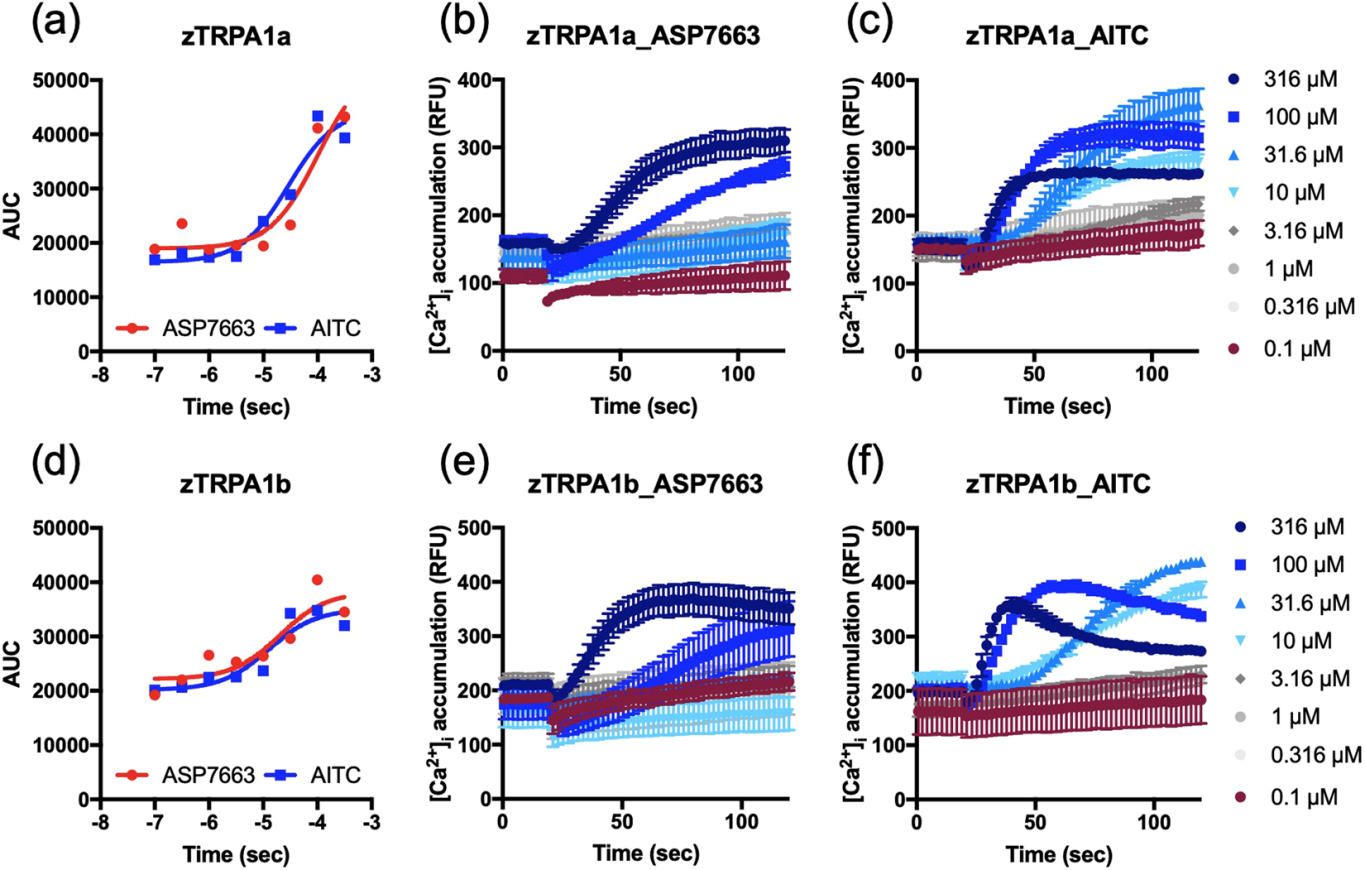
Potency of ASP7663 and AITC in HEK293 cells transfected with zTRPA1a or zTRPA1b. Dose-response curve of ASP7663 and AITC in zTRPA1a-transfected (**a**), and zTRPA1b-transfected (**d**) HEK293 cells. The dose response calcium influx in zTRPA1a-transfected (**b**,**c**), and zTRPA1b-transfected (**e**,**f**) HEK293 cells was measured by Relative Fluorescent Unit (RFU) of ASP7663 and AITC.

We also analyzed dose-response curves of ASP7663 and AITC in zebrafish TRPA1a (zTRPA1a), or zebrafish TRPA1b (zTRPA1b)-transfected HEK293 cells. Dose-response curve of the two agonists (Fig. 4a,d) was plotted based on the area under the curve (AUC) of individual calcium accumulation in Fig. 4b,c,e,f. In line with our previous observation in mTRPA1, ASP7663 and AITC displayed similar potency to zTRPA1a (Fig. 4a, ASP7663: pEC_50_ = 3.9 ± 0.18, 118 µM, n = 7; AITC: EC_50_ = 4.6 ± 0.4, 26.6 µM, n = 4; unpaired t test *p* = 0.1333) and zTRPA1b (Fig. 4d, ASP7663: EC_50_ = 4.0 ± 0.15, 99 µM, n = 7; AITC: EC_50_ = 4.5 ± 0.5, 35.4 µM, n = 4; unpaired t test *p* = 0.2855). Similar slow kinetics with mTRPA1 to 316 µM AITC was observed especially in zTRPA1b (Fig. 4f). Overall, zTPRA1 paralogs had lower pEC_50_ than mTRPA1 to ASP7663 with a statistical significance (One-way ANOVA, F_2,19_ = 18.72, *p* < 0.0001, mTRPA1 vs. zTRPA1a *p* < 0.0001, mTRPA1 vs. zTRPA1b *p* = 0.0002) to AITC with no statistical significance (One-way ANOVA, F_2,10_ = 1.226, *p* = 0.3341, mTRPA1 vs. zTRPA1a *p* = 0.4770, mTRPA1 vs. zTRPA1b *p* = 0.3625). However, we did not find any statistical significance between zTRPA1 paralogs to ASP7663 (zTRPA1a vs. zTRPA1b *p* = 0.9466) or AITC (zTRPA1a vs. zTRPA1b *p* = 0.9749).

### HC-030031 inhibited ASP7663-induced calcium influx of zebrafish TRPA1a and TRPA1b in a dose-dependent manner

**Figure 5:**
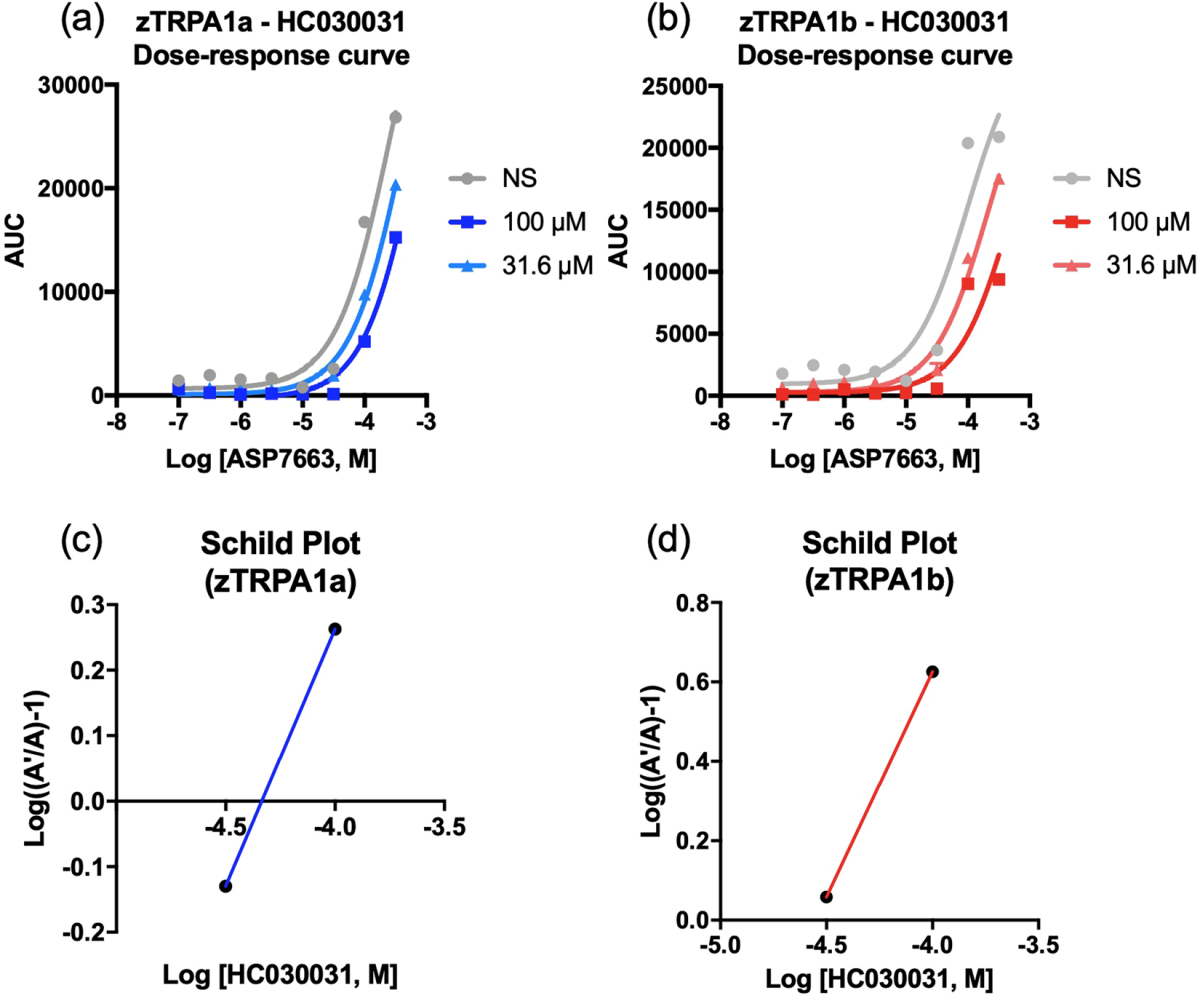
HC-030031 dose-dependently attenuates ASP7663 activation of zTRPA1a and zTRPA1b in transfected HEK293 cells. (**a**,**b**) Dose-response curve of TRPA1 agonist ASP7663 in the absence or presence of HC-030031. Note the shift in the dose-response curve of ASP7663 towards the right with increasing concentration of the antagonist. (**c**,**d**) Schild plot for the HC-030031 at zTRPA1a and zTRPA1b. Representative curves are shown.

After establishing that ASP7663 is a pain-inducing agonist whose effects can be blocked by the TRPA1 antagonist HC-030031, our next step was to pharmacologically characterize these two compounds at the two zebrafish TRPA1 paralogs in transfected HEK 293 cells as we had done for the mTRPA1. Despite the presence of an off-target effect for ASP7663 in non-transfected HEK293 cells (Supplemental Fig. 1), HC-030031 was able to inhibit ASP7663-mediated calcium influx in HEK293 cells transfected with zTRPA1a (pA_2_ = 4.6 ± 0.2, 26.8 µM, n = 3, Fig. 5a,c) or with zTRPA1b (pA_2_ = 4.6 ± 0.3, 27.1 µM, n = 3, Fig. 5b,d individual calcium traces of the dose-response curves are presented in Supplemental Fig. 5). As for the mTRPA1, we noticed that 316 µM HC-030031 could produce an influx of intracellular calcium (Supplemental Fig. 6a–c) in transfected, but not un-transfected cells. Our results suggest that the potency of ASP7663 and efficacy of HC030031 are similar between two zTRPA1 paralogs and that at high concentrations HC-030031 may activate TRPA1.

### TRPA1 agonist-induced nociceptive-like swimming behavior in 5 days post-fertilization (dpf) zebrafish larvae

**Figure 6:**
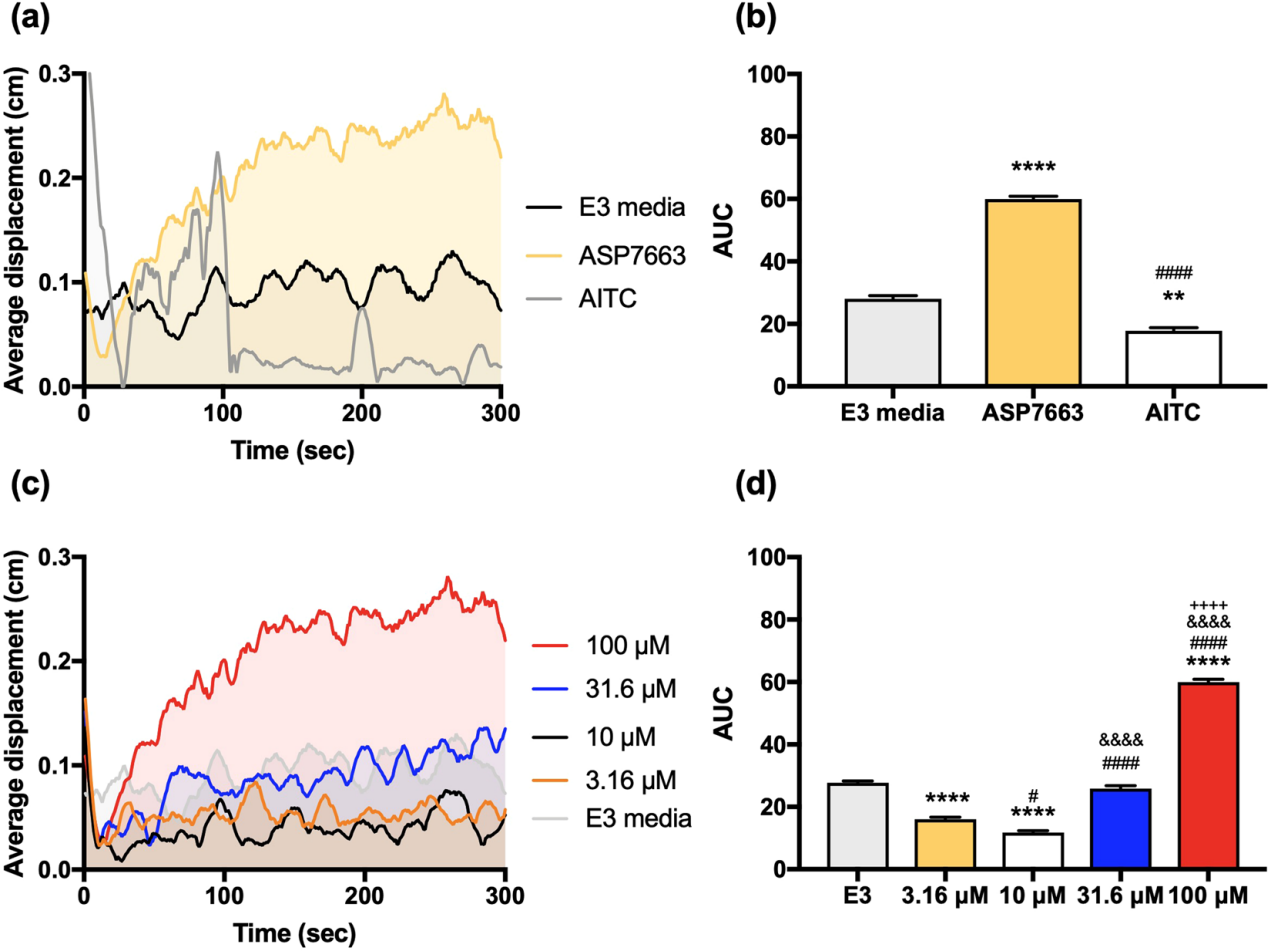
Dose-dependent locomotor response of zebrafish larvae to TRPA1 agonists. (**a**) Displacement graph of average zebrafish locomotion in response to 100 μM ASP7663, 100 μM AITC or E3 media. Zebrafish larvae at 5 dpf are exposed to the TRPA1 agonist at 0 sec. Solid line indicates average distance travelled of 3 biological replicates (n = 8 larvae for each replicate, (**a**)). Area under the curve was further analyzed in (**b**). ((**b**) One-way ANOVA, F_2, 5_ = 491 *p* < 0.0001; E3 vs. ASP7663 ****p < 0.0001; E3 vs. AITC **p < 0.01; ASP7663 vs. AITC ^####^*p* < 0.0001 after Tukey’s multiple comparison). (**c**) Displacement graph of average zebrafish locomotion in response to four series half-log dilutions of TRPA1 agonist ASP7663 and E3 media. Zebrafish larvae at 5 dpf are exposed to the TRPA1 agonist at 0 sec. Solid line indicates average distance travelled of 3 biological replicates (n = 8 larvae for each replicate). (**d**) Area under the curve was further quantified from graph (**c**). ((**d**) One-way ANOVA, F_4, 10_ = 697, p < 0.0001; E3 vs. 3.16 μM: ****p < 0.0001; E3 vs. 10 μM: ****p < 0.0001; E3 vs. 31.6 μM: *p* = 0.429; E3 vs. 100 μM: ****p < 0.0001; 3.16 μM vs. 10 μM: ^#^*p* < 0.05; 3.16 μM vs. 31.6 μM: ^####^*p* < 0.0001; 3.16 μM vs. 100 μM: ^####^*p* < 0.0001; 10 μM vs. 31.6 μM: ^&&&&^*p* < 0.0001; 10 μM vs. 100 μM: ^&&&&^*p* < 0.0001; 31.6 μM vs. 100 μM:^++++^*p* < 0.0001 with Tukey’s post-hoc comparison).

With the acquired knowledge that ASP7663 and HC-030031 pharmacology show little differences between the zTRPA1 paralogs, we next determined whether we could elicit a TRPA1-mediated behavioral response in zebrafish larvae using TRPA1 agonists. We selected 5 dpf zebrafish larvae as they display a repertoire of locomotor behaviors and are easily detected by motion tracking software^25,26^. Additionally, at this age zebrafish will express both TRPA1 paralogs, as previous studies by Prober *et al*. showed that during development zebrafish expressed TRPA1a as early as 72 hours post-fertilization (hpf) and TRPA1b as early as 30 hpf ^21^. Larvae were exposed to two TRPA1 agonists, AITC (100 μM) and ASP7663 (100 μM), and the swimming behavior was tracked. Increased locomotor activity in zebrafish larva in response to TRPA1 agonists including AITC (mustard oil), acrolein and 4-hydroxynonenal may be interpreted as a nocifensive-like escape behavior in response to a noxious stimulus^21^. AITC elicited an acute and rapid swimming behavior immediately upon treatment (Fig. 6a,b; One-way ANOVA, F_2, 5_ = 491 *p* < 0.0001; E3 vs. ASP7663 *p* < 0.0001; E3 vs. AITC *p* < 0.01; ASP7663 vs. AITC *p* < 0.0001 with Tukey’s multiple comparison). In contrast to AITC, ASP7663 induced a slower and more sustained swimming behavior. To determine the dose-dependency of the ASP7663 behavior, larvae were exposed to three additional half-log dilutions of ASP7663 and AUC was analyzed based on the displacement (locomotion) graph (Fig. 6c,d; One-way ANOVA, F_4, 10_ = 697, *p* < 0.0001, E3 vs. 3.16 μM *p* < 0.0001, E3 vs. 10 μM *p* < 0.0001, E3 vs. 31.6 μM *p* = 0.429, E3 vs. 100 μM *p* < 0.0001, 3.16 μM vs. 10 μM *p* < 0.05, 3.16 μM vs. 31.6 μM *p* < 0.0001, 3.16 μM vs. 100 μM *p* < 0.0001, 10 μM vs. 31.6 μM *p* < 0.0001, 10 μM vs. 100 μM *p* < 0.0001, 31.6 μM vs. 100 μM *p* < 0.0001 with Tukey’s multiple comparison). Surprisingly, we observed what appears to be a dose dependent depression of locomotor activity at low ASP7663, which reverses to hyperlocomotor activity around 10 μM to 31.6 μM.

### TRPA1 antagonist pretreatment prevented TRPA1-mediated nocifensive-like locomotor behavior in zebrafish

**Figure 7:**
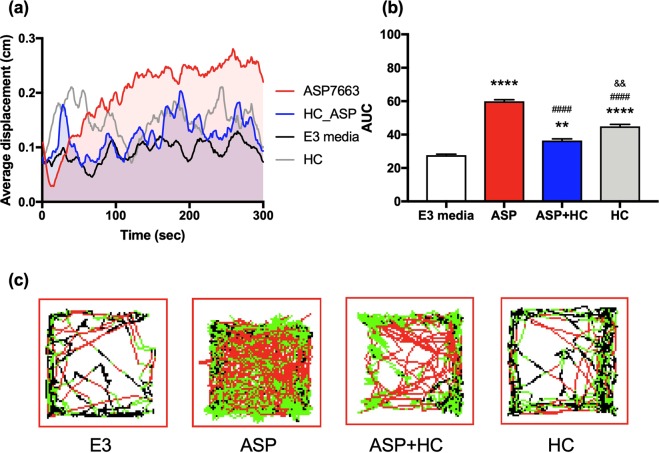
Locomotor response of zebrafish larvae to ASP7663 in presence or absence of HC-030031. (**a**) To determine if TRPA1 antagonists can block ASP7663-mediated channel stimulation, zebrafish were pre-treated with antagonists 10 µM HC-030031 for 20 minutes before challenged with ASP7663. HC-030031 application was able to block ASP7663-mediated locomotor behavior. Solid line indicates average distance travelled of 3 biological replicates (n = 8 larvae for each replicate). (**b**) Area under the curve was further quantified from graph (**a**). (**c**) Representative displacement graph of (**a**,**b**). In the traces, black lines indicate a swimming velocity below 0.6 cm/sec, green lines indicate a swimming velocity between 0.6 cm/sec and 1.0 cm/sec, and red lines indicate a swimming velocity exceeding 1.0 cm/sec. ((**b**) One-way ANOVA, F_3, 8_ = 214.3, p < 0.0001; E3 vs. ASP: ****p < 0.0001; E3 vs. ASP + HC: ** *p* < 0.01; E3 vs. HC: ****p < 0.0001; ASP vs. ASP + HC: ^####^*p* < 0.0001; ASP vs. HC ^####^*p* < 0.000; ASP + HC vs. HC ^&&^*p* = 0.0012 after Sidak’s multiple comparison).

We next assessed if a TRPA1 antagonist could block the ASP7663-induced nocifensive-like behavior in the zebrafish model. Pre-incubation with HC-30031 (10 μM) significantly (ASP7663 vs. ASP + HC, p < 0.0001) inhibited the nocifensive locomotor behavior mediated by ASP7663 (100 μM) administration (Fig. 7a,b; One-way ANOVA, F_3, 8_ = 214.3, *p* < 0.0001, E3 vs. ASP *p* < 0.0001, E3 vs. ASP + HC *p* < 0.01, E3 vs. HC *p* < 0.0001, ASP vs. ASP + HC *p* < 0.0001, ASP vs. HC *p* < 0.000, ASP + HC vs. HC *p* = 0.0012 after Sidak’s multiple comparison). Position tracking of the locomotor behavior of a single representative larva 5 minutes after experimental treatment is further illustrated in Fig. 7c. In the traces, black lines indicate a swimming velocity below 0.6 cm/sec, green lines indicate a swimming velocity between 0.6 cm/sec and 1.0 cm/sec, and red lines indicate a swimming velocity exceeding 1.0 cm/sec. To control for any behavior the antagonists may cause in the zebrafish larvae, we tested zebrafish behavior upon exposure to 10 μM HC-030031 alone. This exposure increased swimming behavior over baseline (E3 vs. HC-030031 *p* < 0.0001). Even though this single treatment of HC-030031 enhanced the swimming behavior, its pre-treatment inhibited TRPA1-induced nocifensive swimming behavior.

## Discussion

Although TRPA1 channels are being considered as a novel target for the development of new chronic pain treatments^27^, there currently are no U.S. Food and Drug Administration approved TRPA1 ligands. Therefore, it is imperative to better characterize TRPA1 pharmacology across different animal species to facilitate drug development and translation of anti-nociceptive TRPA1 drugs for clinical use. Preclinical rodent models are one of the most popular vertebrate models used in drug discovery. One of the biggest hurdles in drug development is showing *in vivo* efficacy. Generally, rodents are used for *in vivo* validation once a lead compound has been generated. Unsurprisingly, much of the physiological and behavioral effects of TRPA1 channels thus far have been established in rodents^6,28^. Only the best ‘hit compounds’ identified in cellular screening assays are moved forward for *in vivo* validation because it would be prohibitive to utilize rodent models to phenotypically screen hundreds of novel anti-nociceptive ‘hits’ due to the time and cost required for rodent studies. The ability to screen drugs in a 96-well plate format using zebrafish larvae highlights the strength of zebrafish as an alternative *in vivo* model for drug discovery. Utilizing zebrafish models has various benefits in the field of drug discovery by providing large phenotype-based screening with its rapid embryonic development^12^. Various behavioral assays of zebrafish have been established for novel drug developments, including for retinal degeneration for example^26^. Zebrafish can also be used to investigate nociceptive-like phenotypes^18,29^ as well as analgesic effects of drugs^15^. A study by Prober *et al*. showed that TRPA1-mediated locomotion can be utilized as distinct characteristic for the nocifensive behavior in zebrafish^21^. In this study, we presented a detailed *in vitro* and *in vivo* pharmacological characterization of mouse and zebrafish TRPA1 using selective agonists and antagonists. Specifically, we demonstrated that activation of TRPA1 increased locomotor behavior of zebrafish in a dose-dependent manner, which was blocked by the TRPA1 antagonist/partial agonist HC-030031. This finding was in line with the ability of HC-030031 to inhibit ASP7663-mediated mechanical sensitivity in mice.

In comparison to mouse TRPA1, zebrafish has two TRPA1 paralogs due to genome duplication, which may have distinct pharmacology and anatomy. Although one study has reported that the zTRAP1a has higher sensitivity to chemical irritant such as AITC than zTRPA1b^30^, our study strongly indicates that the zTRPA1 paralogs acted similarly *in vitro*, as we observed similar potencies for ASP7663 and affinities for HC-030031 at both zTRPA1 paralogs. However, *in vivo*, zTRPA1a expression is limited to the posterior vagal sensory ganglia while zTRPA1b is expressed in all cranial ganglia^21^, which may preclude exogenous agonists from reaching zTRPA1a. Additionally, knockout studies of zTRPA1b reveal loss of sensitivity to the agonist AITC^21,31^, indicating that zTRPA1b is solely responsible for the locomotor behavior.

For the zTRPA1 paralogs, we particularly found that maximal response of ASP7663 was reduced in response to increasing doses of HC-030031 (Fig. 5) while also shifting the EC_50_ values of ASP7663. This finding suggests that HC-030031 may interact with the zTRPA1 paralogs as a competitive or negative allosteric modulator. The Schild slopes for HC-030031 at the zTRPA1 paralogs were lower than 1 (zTRPA1a: 0.84 ± 0.22, n = 3, zTRPA1b: 0.83 ± 0.05, n = 2), which is a textbook definition of competitive interaction^32^. The limitation of the low potency of ASP7663 at the zTRPA1 paralogs is exemplified by our ability to test only two doses of HC-030031 to draw the Schild plot, where it is recommended to test five different antagonist concentrations. Having only two concentration data points limits our ability to definitively calculate pA2 values for the antagonist and determine the slope. This is in line with a potential limitation of using ASP7663 in zebrafish given that the potency of ASP7663 and affinity for HC-030031 was lower at the zTRPA1 paralogs in comparison to mTRPA1. Discovery or development of stronger TRPA1-selective agonist, such as crotalphine^33^, could be beneficial in replacing ASP7663 for future screening efforts with a larger range of detection and will help provide better quality pA_2_ calculation.

We further suggest that the observed hyperlocomotion in zebrafish is TRPA1-mediated. First, TRPA1-mediated hyperlocomotion has been previously reported by others^16,21^. Second, both AITC, although briefly, and ASP7663 produce hyperlocomotion, and both agonists increase calcium signaling *in vitro* via TRPA1. Third, ASP7663-induced hyperlocomotion was attenuated by administration of HC-030031. Fourth, HC-030031 increases calcium release in HEK293 cells at a high concentration, and a high dose of HC-030031 *in vivo* produces significant hyperlocomotion albeit weaker than ASP7663 (Fig. 7b). An unexpected observation, however, was that at low doses (3.16 and 10 μM) ASP7663 induced hypolocomotion in zebrafish (Fig. 6c,d), but hyperlocomotor activity at higher concentration (31.6 and 100 μM). We currently do not have an explanation for the hypolocomotion, but this may be a potent off-target effect in zebrafish (Supplemental Fig. 1) that is masked by hyperlocomotion once TRPA1 is activated. Furthermore, our observation that the TRPA1 antagonist HC-030031 may have some partial agonistic effects is novel.

The nocifensive behavior in zebrafish was initially demonstrated using mustard oil^34^, which contains the TRPA1 agonist AITC. AITC has been shown to also activate TRPM8^23^ and TRPV1^24^, and thus it would be suboptimal to use this agonist to screen for TRPA1-selective antagonists. However, given the fact that zebrafish lack TRPM8 channels^29^, selectivity may be less of a concern in this species. A different concern is that AITC activation of TRPA1 channel has been shown to lead to rapid TRPA1 desensitization and internalization^35–37^, a feature we observe also in the calcium assay for this agonist (Fig. 4f, see particularly at 316 µM) and in zebrafish (see Fig. 6a at 100 seconds AITC). In our hands, AITC has similar potency with the TRPA1-selective agonist ASP7663, but we found no indication of rapid desensitization for ASP7663. The lack of rapid desensitization by ASP7663 was a reason for us to choose this agonist to investigate the pharmacological profiles of mouse and zebrafish TRPA1 channels in both *in vivo* model systems. The *in vitro* desensitization profile of AITC may correlate with its *in vivo* profile, where a short initial bout of AITC-induced hyperlocomotion is followed by a rapid decline in locomotor activity. In contrast, application of ASP7663 in zebrafish larvae showed more prolonged agonist-mediated locomotor behavior than AITC (Fig. 5a,b). At the high concentration of HC-030031 at which we observe calcium influx, the kinetic profile resembles that of ASP7663 and similarly HC-030031 produces a moderate but persistent increase in locomotor activity, suggesting that HC-030031 does not rapidly desensitize zTRPA1.

The TRPA1 agonist ASP7663 also produced mechanical hypersensitivity in mice (Fig. 3), a widely used nociception model, and was blocked by the TRPA1 antagonist HC-030031. This finding mimics our observations using the same agonist and antagonist in zebrafish locomotor behavior (Figs 6 and 7). A study by Stevens *et al*. previously found that HC-030031 was able to inhibit locomotor responses in zebrafish larvae induced by the TRPA1 agonist acrolein^16^ and further support the face validity of the zebrafish model.

In addition to the finding that ASP7663-induced nocifensive-like behavior, we found similarities in ASP7663-mediated calcium influx from both zebrafish and mouse TRPA1 channels. In mTRPA1, ASP7663 dose-dependently induced calcium signaling, and TRPA1 antagonists attenuated this influx. Of the three antagonists, HC-030031 and TCS-5861528 had lower antagonist-channel affinities compared to A-967076 (Fig. 2). This is in agreement with published characterization of A-967076 interaction with rat TRPA1 in which the antagonist has an IC_50_ of 0.289 µM, and A-967076 was found to be about 25-fold stronger than HC-030031^38^.

We also noted some off-target effects in our HEK293 cells; specifically, ASP7663 showed calcium influx at high concentration (316 µM) in non-transfected HEK293 cells. It is possible that the agonist may interact with endogenous calcium channels in HEK293 cell^39^. Nonetheless, it is important to note that the potency of ASP7663 was much lower in non-transfected HEK293 compared with HEK293 transfected with mTRPA1 and we were able to block the intracellular calcium release using TRPA1 antagonists for both the mTRPA1 and the zTRPA1 paralogs. The existence of an off-target effect, however, may explain why we did not obtain a perfect slope for the Schild plots for the TRPA1 antagonists.

## Conclusions

Overall, our critical analysis of currently commercialized TRPA1 agonists and antagonists in mouse TRPA1 and the two zebrafish TRPA1 paralogs have found similarities in line with previous published observations particularly in agonist-mediated hyperalgesia in mice and hyperlocomotion in zebrafish. However, our study revealed several novel findings. First, the kinetics of calcium release and zebrafish hyperlocomotion were not identical between TRPA1 agonists. Second, both HEK293 cells and zebrafish may exhibit non-TRPA1 targets that respond to the TRPA1 agonist ASP7663. Third, the TRPA1 antagonist HC-030031 may activate TRPA1 *in vitro* and *in vivo* at high enough concentrations. Finally, the potency of TPRA1 agonists and antagonists appears to be stronger for mTRPA1 than the zTRPA1 paralogs. Taken together, we propose that TRPA1-mediated hyperlocomotion in zebrafish has the potential to be a useful phenotypic assay to for TRPA1 drug screening and discovery. As for all compound screening, secondary assays will still be required to assess channel-ligand pharmacology including receptor desensitization, potential off-target effects of a ligand, and an ability of a ligand to serve as an antagonist, partial agonist, or a full agonist.

## Methods

### Animals

#### Zebrafish (*Danio rerio*)

Wild-type zebrafish of the AB line were utilized for all behavioral experiments. Adult and larval zebrafish were maintained on a 14 hr/10 hr light/dark cycle. They were maintained and bred using standard procedure https://zfin.org/zf_info/zfbook/zfbk.html. Larval zebrafish were reared until 5 days post-fertilization (dpf) in E3 media https://zfin.org/zf_info/zfbook/chapt1/1.3.html in an incubator at 28 °C. E3 media was changed daily, and healthy embryos were kept for experiments.

#### Mice (Mus musculus)

We utilized WT C57BL/6 mice purchased from Envigo (Indianapolis, IN, USA). Total sixteen adult male mice (8–9 weeks, 22 ± 2 g) were housed in four ventilated Plexiglas cages (4 mice per cage). Four mice were tested in each group. Mice are maintained at temperature (21 °C) in an animal housing facility accredited by the Association for Assessment and Accreditation of Laboratory Animal Care with a reversed 12-hour dark-light cycle (lights off at 1000, lights on at 2200). This study was carried out in accordance with the recommendations of the National Institutes of Health Guide for the Care and Use of Laboratory Animals. The protocol (#1201000592 by Y.F.L. for Zebrafish, and #1605001408 by R.M.vR for mice) was approved by the Purdue University Institutional Animal Care and Use Committee.

### Cell culture

HEK293 cells (#CRL-1573, ATCC, VA, USA) were cultured in DMEM media (#11995-065, Sigma-Aldrich, MO, USA) with 10% Fetal Bovine Serum (#F0926-500ML, Thermo Fisher, MA, USA). The cells were maintained in a 37 °C incubator with consistent 5% CO_2_. They were seeded in a clear 6 well flat bottom cell culture plates (#07-200-83, Corning ®, Thermo Fisher) with 500,000 cells/2 ml/well for transfection, in serum-free Opti-MEM (#31985070, Gibco®, Thermo Fisher). These cells were transfected with pcDNA3.1-mTRPA1 (#MR227099, OriGene, MD, USA), pcDNA3.1-zTRPA1a, or pcDNA3.1-zTRPA1b (a gift from Dr. David Prober) using X-tremeGENE^TM^ 9 (#6365809001, Sigma-Aldrich). After 24 hours, the transfected cells were dislodged with trypsin, resuspended in Opti-MEM, and seeded 25,000 cells/25 µl/well in 384-well black polystyrene microplates (#82051-296, VWR, PA, USA) for testing calcium signaling the following day. Experiments were carried out with the approval of the Institutional Biohazard Committee (#13-013-16).

### FLIPR calcium signaling assay

Twenty-five µl calcium sensitive Fluorescent Imaging Plate Reader (FLIPR) Calcium 6 assay dye (#R8190, Molecular Devices, CA, USA) was added to each well of a 384-well plate containing HEK293 cells transiently expressing mTRPA1, zTRPA1a, and zTRPA1b, respectively. The cells were incubated for an hour prior to the recording of intracellular calcium levels in a FlexStation3 Multi-Mode Microplate Reader (#R8190, Molecular Devices) as previously described^40^. All compounds were diluted in calcium buffer made with 1x HBSS (#14025-092, Thermo Fisher), 20 mM HEPES (#15630-080, Thermo Fisher), and 2.5 mM Probenecid (#P8761, Sigma-Aldrich). For agonist studies, ASP7663 (#5178, Tocris, Bristol, UK) was diluted in 1% DMSO-containing calcium buffer at desired concentrations and was added during the recording. The TRPA1 antagonists HC-030031 (#2896, Tocris), TCS-5861528 (#3938, Tocris), and A-967079 (#4716, Tocris) were diluted in calcium buffer, and 5 µl of 10x solutions were added 10 minutes prior to the recording. The same amount of DMSO (1%) was added to all control groups to maintain similar DMSO levels with the experimental groups. During the assay, the cells were challenged by a 5x ASP7663 solution or 1% DMSO solution at the 20 seconds time point, and both solutions were diluted in calcium buffer. Relative fluorescence units (RFU) were measured for 120 seconds period by the Flexstation. Area under the curve (AUC) was further evaluated from individual calcium influx data (Supplemental Figs 2 and 4) to plot does-response curve of antagonists. For each specific antagonist concentration we plotted Log(dose ratio-1) against the antagonist concentration in a Schild plot. The dose ratio (A’/A) equals the EC_50_ of the agonist (ASP7665) in the presence of a concentration of the antagonist (A’) divided by the EC_50_ of the agonist in the absence of antagonist (A). The x-intercept of the Schild plot was used to identify the antagonist-receptor affinity (pA_2_).

### Von Frey Test

Von Frey filaments (2.44 (0.04 g)–4.31 (2 g)) and a grid platform (#58011 and #57816, Stoelting, WI, USA) were used to test mechanical hypersensitivity of mice. A modified up-and-down method was utilized as previously described^41^. Immediately after measuring the baseline, the mice were injected with 100 mg/kg HC-030031 (p.o., diluted in 5% DMSO, 0.5% methylcellulose in saline), whereas the control group mice were injected with the appropriate vehicle solution (5% DMSO, 0.5% methylcellulose in saline). Thirty minutes after the first injection, mice were systemically administered 1 mg/kg ASP7663 (i.p., diluted in 1% DMSO in saline), or vehicle (1% DMSO in saline). Drug-induced mechanical hypersensitivity was measured 30 minutes after the second injection. Pre- and post-drug responses were represented as mean ± SEM.

### Zebrafish Locomotor Tracking Assay

In the behavioral experiments, one 5 dpf zebrafish was placed in 250 μL of fish media per well of a 96-well plate (UNIPLATE Collection and Analysis Microplate, 96-Well 7701-1651). For each experimental condition, a group of 8 larvae (1 column) was used. Larvae were acclimated to the wells for 300 seconds after which an 8-channel pipette was used to simultaneously dispense 250 μL of 2x concentration of the TRPA1 agonist ASP7663 in fish media into each well. Locomotor activity was subsequently tracked and quantified (as distance travelled) for 300 additional seconds utilizing the Zebrabox system from ViewPoint Behavior Technology (Civrieux, France). For TRPA1 antagonist testing, larvae in the 96-well plate were pretreated with 1x concentration of antagonist in 250 μL E3 media for 20 minutes. Then, 250 μL of 2x TRPA1 agonist was added directly to this solution, and the resulting behavior was recorded as described above.

### Statistical Analysis

All data are presented as means ± standard error of the mean (SEM). For our *in vitro* statistical analysis, AUC of calcium influx (RFU) was measured to plot dose-response curves, which were further analyzed by nonlinear regression analysis. Mean RFU of the first 10 seconds was used as a baseline of the AUC. EC_50_ values of the dose-response curves were further used to plot Schild curves, and the curves were analyzed with linear regression analysis. For the mice von Frey test, mechanical thresholds were analyzed with two-way ANOVA followed by a Bonferonni test for multiple comparison analysis. For the zebrafish behavior test, the data from the first five seconds following drug administration were excluded, as zebrafish displacement during that period was highly influenced by liquid dispensing. AUC of all curves were measured and analyzed by one-way ANOVA. The post-hoc analysis was conducted with the Tukey’s or Dunnett’s multiple comparisons unless it stated otherwise. A value of y = 0 in the graph was used as a baseline of the AUC. For our time-course average displacement graph, we used 2^nd^ degree polynomial smoothing for a better representation; however, we used data before smoothing for all statistical analysis and quantification. All *in vitro* and *in vivo* data were evaluated using GraphPad Prism 7 (GraphPad Software, La Jolla, CA) unless it stated otherwise.

## Supplementary information

Supplementary info

## Data Availability

All materials, data and associated protocols will be promptly made available to readers upon request without undue qualifications in material transfer agreements.

## References

[1] Dowell D, Haegerich TM, Chou R (2016). CDC Guideline for Prescribing Opioids for Chronic Pain - United States, 2016. MMWR Recomm Rep.

[2] Nassini R, Materazzi S, Benemei S, Geppetti P (2014). The TRPA1 channel in inflammatory and neuropathic pain and migraine. Rev Physiol Biochem Pharmacol.

[3] Chen J, Hackos DH (2015). TRPA1 as a drug target–promise and challenges. Naunyn Schmiedebergs Arch Pharmacol.

[4] Tominaga, M. Nociception and TRP channels. *Handb Exp Pharmacol*, 489–505, 10.1007/978-3-540-34891-7_29 (2007). 10.1007/978-3-540-34891-7_29 17217075

[5] Story GM (2003). ANKTM1, a TRP-like channel expressed in nociceptive neurons, is activated by cold temperatures. Cell.

[6] Petrus M (2007). A role of TRPA1 in mechanical hyperalgesia is revealed by pharmacological inhibition. Mol Pain.

[7] Corey DP (2004). TRPA1 is a candidate for the mechanosensitive transduction channel of vertebrate hair cells. Nature.

[8] Bautista DM (2005). Pungent products from garlic activate the sensory ion channel TRPA1. Proc Natl Acad Sci USA.

[9] Due MR (2014). Acrolein involvement in sensory and behavioral hypersensitivity following spinal cord injury in the rat. J Neurochem.

[10] McNamara CR (2007). TRPA1 mediates formalin-induced pain. Proc Natl Acad Sci USA.

[11] Eid SR (2008). HC-030031, a TRPA1 selective antagonist, attenuates inflammatory- and neuropathy-induced mechanical hypersensitivity. Mol Pain.

[12] Zon LI, Peterson RT (2005). *In vivo* drug discovery in the zebrafish. Nat Rev Drug Discov.

[13] Bretaud S (2007). A choice behavior for morphine reveals experience-dependent drug preference and underlying neural substrates in developing larval zebrafish. Neuroscience.

[14] Mathur P, Guo S (2011). Differences of acute versus chronic ethanol exposure on anxiety-like behavioral responses in zebrafish. Behav Brain Res.

[15] Curtright A (2015). Modeling nociception in zebrafish: a way forward for unbiased analgesic discovery. PLoS One.

[16] Stevens JS (2018). Zebrafish Locomotor Responses Reveal Irritant Effects of Fine Particulate Matter Extracts and a Role for TRPA1. Toxicol Sci.

[17] Arenas OM (2017). Activation of planarian TRPA1 by reactive oxygen species reveals a conserved mechanism for animal nociception. Nat Neurosci.

[18] Taylor JC (2017). A novel zebrafish-based model of nociception. Physiol Behav.

[19] Gonzalez-Nunez V, Jimenez Gonzalez A, Barreto-Valer K, Rodriguez RE (2013). *In vivo* regulation of the mu opioid receptor: role of the endogenous opioid agents. Mol Med.

[20] Marron Fdez de Velasco E, Law PY, Rodriguez RE (2009). Mu opioid receptor from the zebrafish exhibits functional characteristics as those of mammalian mu opioid receptor. Zebrafish.

[21] Prober DA (2008). Zebrafish TRPA1 channels are required for chemosensation but not for thermosensation or mechanosensory hair cell function. J Neurosci.

[22] Kojima R (2014). Effects of novel TRPA1 receptor agonist ASP7663 in models of drug-induced constipation and visceral pain. Eur J Pharmacol.

[23] Janssens, A. *et al*. Definition of two agonist types at the mammalian cold-activated channel TRPM8. *Elife* **5**, 10.7554/eLife.17240 (2016). 10.7554/eLife.17240 PMC4985286 27449282

[24] Gees M (2013). Mechanisms of transient receptor potential vanilloid 1 activation and sensitization by allyl isothiocyanate. Mol Pharmacol.

[25] Kalueff AV (2013). Towards a comprehensive catalog of zebrafish behavior 1.0 and beyond. Zebrafish.

[26] Ganzen, L., Venkatraman, P., Pang, C. P., Leung, Y. F. & Zhang, M. Utilizing Zebrafish Visual Behaviors in Drug Screening for Retinal Degeneration. *Int J Mol Sci* **18**, 10.3390/ijms18061185 (2017). 10.3390/ijms18061185 PMC5486008 28574477

[27] Moran MM, McAlexander MA, Biro T, Szallasi A (2011). Transient receptor potential channels as therapeutic targets. Nat Rev Drug Discov.

[28] Gerlai R (2010). High-throughput behavioral screens: the first step towards finding genes involved in vertebrate brain function using zebrafish. Molecules.

[29] Chen S, Chiu CN, McArthur KL, Fetcho JR, Prober DA (2016). TRP channel mediated neuronal activation and ablation in freely behaving zebrafish. Nat Methods.

[30] Oda M, Kurogi M, Kubo Y, Saitoh O (2016). Sensitivities of Two Zebrafish TRPA1 Paralogs to Chemical and Thermal Stimuli Analyzed in Heterologous Expression Systems. Chem Senses.

[31] Esancy, K. *et al*. A zebrafish and mouse model for selective pruritus via direct activation of TRPA1. *Elife* **7**, 10.7554/eLife.32036 (2018). 10.7554/eLife.32036 PMC5912907 29561265

[32] Colquhoun D (2007). Why the Schild method is better than Schild realised. Trends Pharmacol Sci.

[33] Bressan E (2016). Crotalphine desensitizes TRPA1 ion channels to alleviate inflammatory hyperalgesia. Pain.

[34] Eilers H (2010). Pungent general anesthetics activate transient receptor potential-A1 to produce hyperalgesia and neurogenic bronchoconstriction. Anesthesiology.

[35] Raisinghani M (2011). Activation characteristics of transient receptor potential ankyrin 1 and its role in nociception. Am J Physiol Cell Physiol.

[36] Akopian AN, Ruparel NB, Jeske NA, Hargreaves KM (2007). Transient receptor potential TRPA1 channel desensitization in sensory neurons is agonist dependent and regulated by TRPV1-directed internalization. J Physiol.

[37] Kistner K (2016). Systemic desensitization through TRPA1 channels by capsazepine and mustard oil - a novel strategy against inflammation and pain. Sci Rep.

[38] Chen J (2011). Selective blockade of TRPA1 channel attenuates pathological pain without altering noxious cold sensation or body temperature regulation. Pain.

[39] Bugaj V (2005). Functional properties of endogenous receptor- and store-operated calcium influx channels in HEK293 cells. J Biol Chem.

[40] van Rijn RM, Harvey JH, Brissett DI, DeFriel JN, Whistler JL (2013). Novel screening assay for the selective detection of G-protein-coupled receptor heteromer signaling. J Pharmacol Exp Ther.

[41] van Rijn RM, Brissett DI, Whistler JL (2012). Emergence of functional spinal delta opioid receptors after chronic ethanol exposure. Biol Psychiatry.

