# Supplementary material for: A critical evaluation of TRPA1-mediated locomotor behavior in zebrafish as a screening tool for novel anti-nociceptive drug discovery
Source: Sci Rep. 2019 Feb 20;9:2430. doi: 10.1038/s41598-019-38852-9 (PMC6382835; doi:10.1038/s41598-019-38852-9)
Supplement: Supplementary file 1 — Supplementary info [file 41598_2019_38852_MOESM1_ESM.pdf]

Title page

Title: A critical evaluation of TRPA1-mediated locomotor behavior in zebrafish as a screening tool for novel anti-nociceptive drug discovery

Authors: Mee Jung Ko[1,3,4†], Logan C. Ganzen[2,3,4†], Emre Coskun[2], Arbaaz A. Mukadam[1], Yuk Fai Leung[2,3,4,5,6], Richard M. van Rijn[1,3,4,5]

Supplementary information

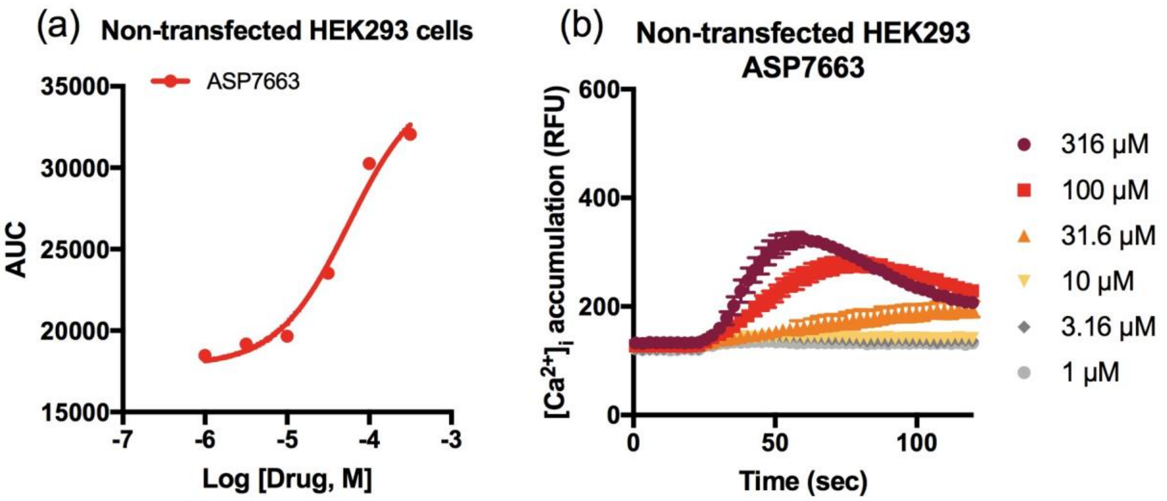

Supplemental Figure 1. Potential off-target effect of ASP7663 in non-transfected HEK293 cells (a) Dose-response curve of ASP7663 in non-transfected HEK293 cells (ASP7663: pEC_{50} = 4.27 ± 0.03, n=3). Relative Fluorescent Unit (RFU) of ASP7663 dose response calcium influx in non-transfected HEK293 **(b)**. **(a)** was measured by area under the curve (AUC) of the RFU.

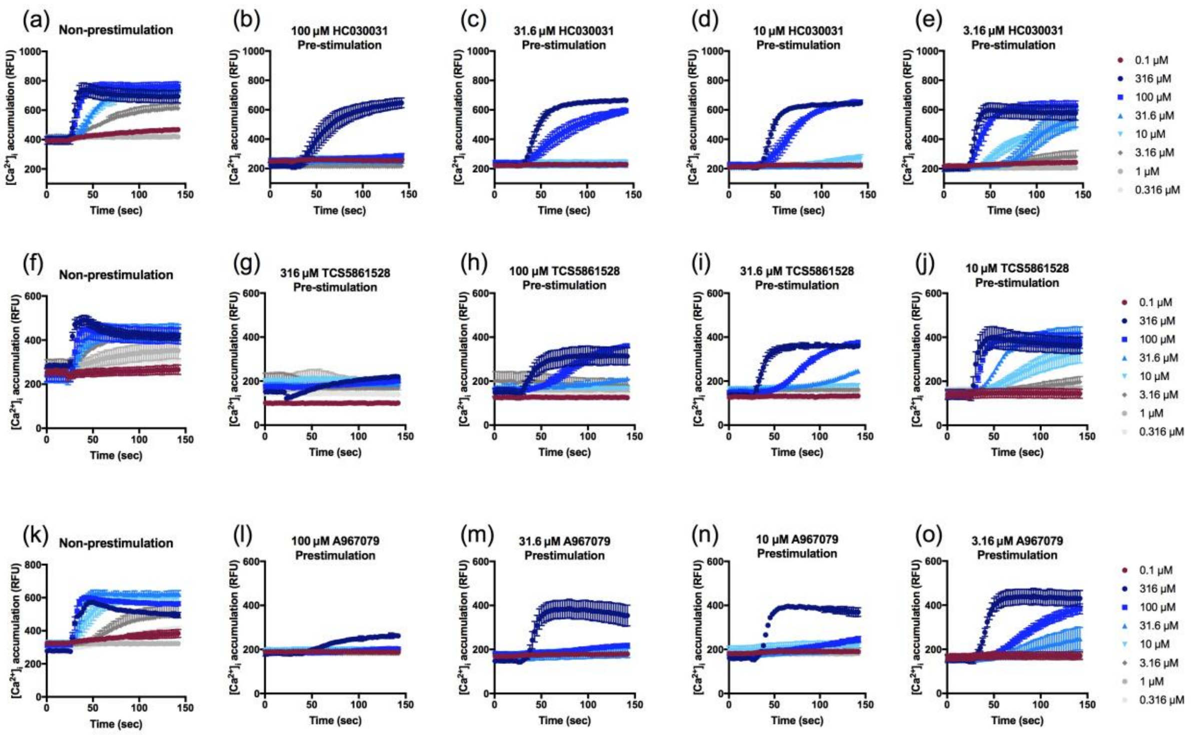

Supplemental Figure 2. Calcium influx to TRPA1 antagonists in HEK293 cells transiently expressing mTRPA1. (a, f, k) Intracellular calcium levels of HEK293 cells transiently expressing mTRPA1 to ASP7663 with half-log dilutions. **(b-e)** ASP7663 dose-response calcium influx to HC-030031 with half-log dilutions. **(g-j)** ASP7663 dose-response calcium influx to TCS-5861528 with half-log dilutions. ASP7663 dose-response calcium influx to A-967079 with half-log dilutions. The AUC of the RFU was further analyzed in Figure 2.

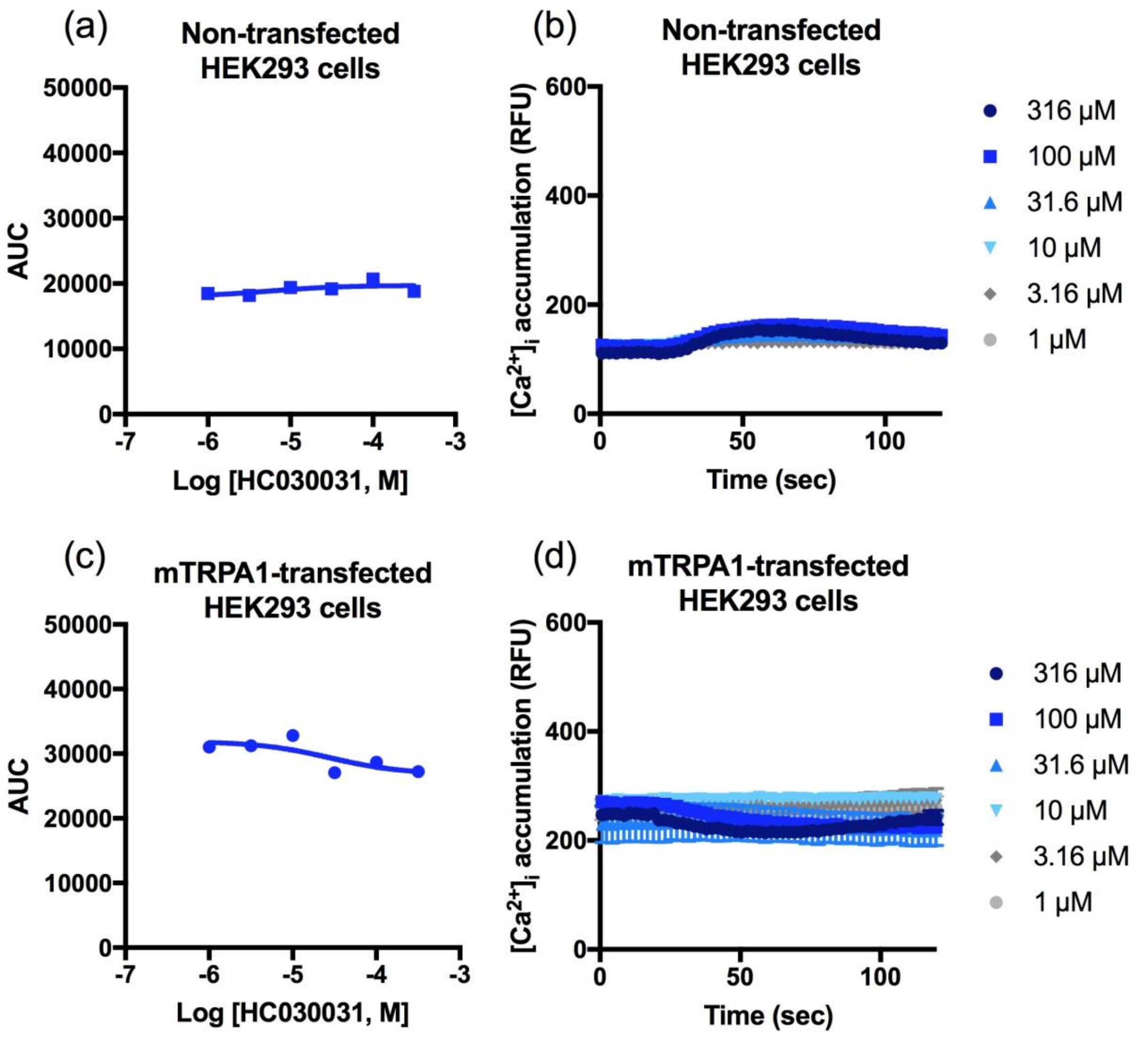

Supplemental Figure 3. Calcium influx to HC-030031 in non-transfected or mTRPA1 transfected HEK293 cells. Dose response intracellular calcium levels of HC030031 in non-transfected HEK293 cells **(a)** and mTRPA1-transfected HEK293 cells **(c).** Relative Fluorescent Unit (RFU) of HC030031 dose response calcium influx in non-transfected HEK293 **(b, d). (a, c)** were measured by area under the curve (AUC) of the RFU. (mTRPA1: pIC_{50} = 5.77 ± 0.21, n=4).

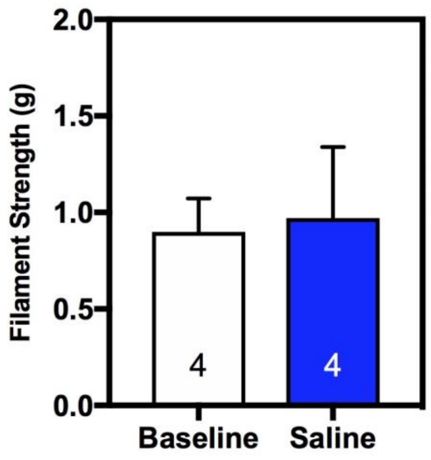

Supplemental Figure 4. Mechanical hypersensitivity was not changed by saline injection in C57BL/6 mice. Mechanical sensitivity was measured in C57BL/6 mice pre- and post-saline administration (n=4 per treatment) in response to von Frey filament stimulation. (Unpaired t-test, p=0.8692).

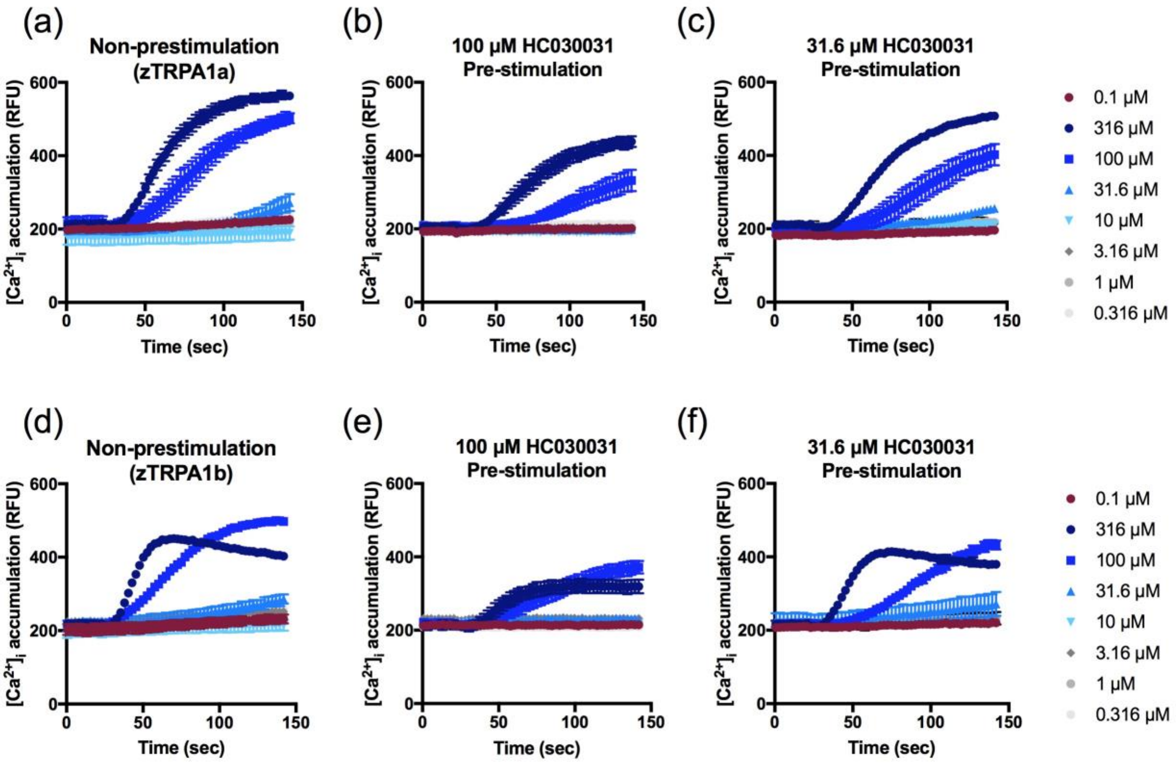

Supplemental Figure 5. Calcium influx to HC-030031 in HEK293 cells transiently expressing zTRPA1a and zTRPA1b. (a, b) Intracellular calcium levels of HEK293 cells transiently expressing zTRPA1a and zTRPA1b respectively to ASP7663 with half-log dilutions. **(b,c)** ASP7663 dose-response calcium influx in zTRPA1a to HC-030031 with a full-log dilution. **(e,f)** ASP7663 dose-response calcium influx in zTRPA1b to HC-030031 with a full-log dilution. The AUC of the RFU was further analyzed in Figure 4.

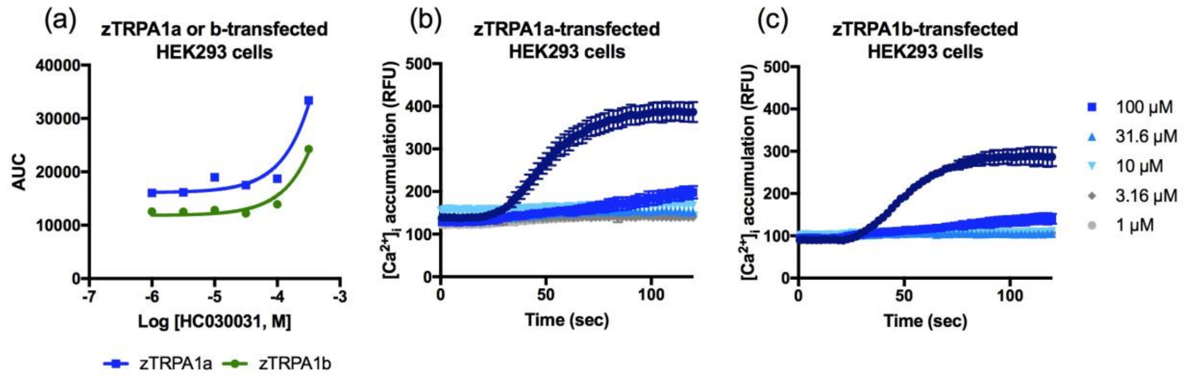

Supplemental Figure 6. HC030031 works as weak agonist at high concentration. (a) Dose-response curve of HC030031 in zTRPA1a-transfected, and zTRPA1b-transfected (Green) HEK293 cells (zTRPA1a: pEC_{50} = 2.85 ± 0.19, n=4; zTRPA1b: Non-determinant, n=3). Relative Fluorescent Unit (RFU) of HC030031 dose response calcium influx in zTRPA1a-transfected **(b)**, and zTRPA1b-transfected **(c)** HEK293 cells. **(a)** was measured by area under the curve (AUC) of the RFU.
